# Platelet P2Y12 inhibitor use and risk of colorectal cancer incidence and colorectal cancer-related mortality: a Swedish registry-based cohort study

**DOI:** 10.1186/s12876-026-05422-z

**Published:** 2026-10-12

**Authors:** Michael Fuchs, Jane Ha, Jane Yan, Matteo Bottai, Mingyang Song, Tracey G. Simon, Weimin Ye, Andrew T. Chan, Jonas F. Ludvigsson

**Affiliations:** 1 Division of Gastroenterology, Department of Medicine, Brigham and Women’s Hospital and Harvard Medical School, Boston, MA USA; 2 Clinical and Translational Epidemiology Unit, Massachusetts General Hospital and Harvard Medical School, Boston, MA USA; 3 Division of Gastroenterology, Department of Medicine, Massachusetts General Hospital and Harvard Medical School, Boston, MA USA; 4 Division of Biostatistics, Institute of Environmental Medicine, Karolinska Institutet, Stockholm, Sweden; 5 Department of Epidemiology, Harvard T.H. Chan School of Public Health, Boston, MA USA; 6 Department of Nutrition, Harvard T.H. Chan School of Public Health, Boston, MA USA; 7 Department of Medical Epidemiology and Biostatistics, Karolinska Institutet, Stockholm, Sweden; 8 Channing Division of Network Medicine, Brigham and Women’s Hospital and Harvard Medical School, Boston, MA USA; 9 Broad Institute of MIT and Harvard, Cambridge, MA USA; 10 Department of Immunology and Infectious Diseases, Harvard T.H. Chan School of Public Health, Boston, MA USA; 11 Department of Pediatrics, Orebro University Hospital, Orebro, Sweden; 12 Department of Medicine, Columbia University College of Physicians and Surgeons, New York, NY USA

**Keywords:** Colorectal cancer, P2Y12 inhibitor, Antiplatelet, Chemoprevention

## Abstract

**Background:**

Low-dose aspirin use is associated with reduced CRC incidence and CRC-related mortality. A similar association with P2Y12 inhibitor use remains uncertain. We investigated whether the use of P2Y12 inhibitors was associated with CRC incidence and CRC-related mortality among individuals diagnosed with atherosclerotic cardiovascular disease (ASCVD).

**Methods:**

From the ESPRESSO cohort, we identified 334,882 patients diagnosed with ASCVD between 2006 and 2021. We identified patients who initiated P2Y12 inhibitors ≥ 84 consecutive Defined Daily Doses (cDDDs). We estimated the risk of incident CRC and CRC mortality according to P2Y12 inhibitor use using Cox proportional-hazards regression models adjusting for potential confounders including comorbidities and regular aspirin use.

**Results:**

We documented 4,015 incident CRC cases and 2,610 CRC-related deaths during a median follow-up of 5.1 years (IRQ, 2.1–9.0 years). We did not observe an association between P2Y12 inhibitor use and CRC incidence. In contrast, a longer duration of P2Y12 inhibitor use was associated with lower CRC-related mortality. Compared to non-users, multivariable-adjusted hazard ratio (HR) for CRC mortality were 0.73 (95% CI, 0.60–0.89) in patients with 336–671 cDDDs of P2Y12 inhibitor and 0.81 (95% CI, 0.68–0.97) in those with ≥672 cDDDs. Among patients with incident CRC diagnosis, subsequent initiation of P2Y12 inhibitor after CRC diagnosis was associated with significantly lower CRC mortality (HR 0.42; 95% CI, 0.29–0.60).

**Conclusions:**

P2Y12 inhibitor use was not associated with risk of incident CRC but was significantly inversely associated with CRC mortality, which appeared to be due to improved survival among individuals who initiate P2Y12 inhibitors after CRC diagnosis. These findings merit further study given the observational nature of the analysis and potential for residual confounding.

## Background

Colorectal cancer (CRC) is the third most common cancer and the second leading cause of cancer-related death worldwide [1]. Numerous observational and randomized studies demonstrate a protective association between daily low-dose aspirin use and CRC incidence and CRC-related mortality [2–5]. As a result, in 2016 the United States Preventive Services Taskforce (USPSTF) recommended daily low-dose aspirin for the primary prevention of CRC in adults aged 50 to 59 years who have a 10% or greater 10-year cardiovascular risk and no risk of bleeding [6].

There remains debate over the mechanism by which aspirin exerts a protective effect against CRC. Aspirin irreversibly inhibits both prostaglandin synthase (PTGS)-1 (also known as cyclooxygenase (COX)-1), which is constitutively expressed in platelets and gastric epithelial cells, as well as PTGS-2, (also known as COX-2), which is overexpressed in CRC cells. The production of prostaglandin E2 by PTGS-2 is thought to be critical for CRC carcinogenesis and progression [7]. Although aspirin inhibits PTGS-2, it is unclear if aspirin at low doses sufficiently inhibits PTGS-2 in humans [8]. Low-dose aspirin, however, completely inhibits platelet COX-1 and effectively inhibits prostaglandin synthesis in the colon [7]. In addition, activated platelets release molecular factors that potentially induce PTGS-2 expression in gastrointestinal mucosa [9]. Platelet inhibition has thus been proposed to contribute to aspirin’s chemoprotective effect [7].

If platelet inhibition underlies the protective benefit of aspirin against CRC, other antiplatelet drugs without effects on PTGS-1 or PTGS-2 may also be inversely associated with CRC risk. The class of P2Y12 inhibitors, which includes clopidogrel, prasugrel, and ticagrelor, act antagonistically on the platelet adenosine diphosphate receptor P2Y12 to prevent platelet aggregation [10]. Similar to aspirin, P2Y12 inhibitors are used in the treatment of atherosclerotic cardiovascular disease (ASCVD) [11], but their effect on cancer is unclear. Previously, *Rodríguez-Miguel et al (2018*) found clopidogrel monotherapy was associated with a significantly reduced incidence of CRC [12]. To this end, we aimed to investigate whether use of P2Y12 inhibitors was associated with CRC incidence and related mortality among individuals diagnosed with ASCVD.

## Methods

### Study population

Between October 2015 and April 2017, we collected data on gastrointestinal histopathology from all 28 pathology departments in Sweden. We retrieved biopsy reports from 2.1 million unique individuals in Sweden dated between 1965 and 2017. Index individuals with histopathology reports were subsequently matched to up to five reference individuals from the Swedish Total Population Register according to age, sex, calendar year (of biopsy), and county of residence [13]. In total, the ESPRESSO cohort (Epidemiology Strengthened by histopathology Reports in Sweden) contains data on 13.0 million individuals [14].

The Swedish Prescribed Drug Register (PDR) was established in July of 2005, at which time, data on dispensed drugs became available. We restricted our cohort to individuals with ≥1 year of data from the Swedish Prescribed Drug Register and started follow-up after July 1, 2006. The PDR includes data on dispensed medications, as opposed to issued prescriptions, thereby better reflecting true drug exposure. The PDR does not contain data on hospital-administered drugs and patient identity data is available in more than 99.7% of all drug records [15].

We identified individuals with a diagnosis of ASCVD after the ESPRESSO cohort entry (first biopsy date or matched date) between July 2006 and November 2021 using relevant International Classification of Diseases (ICD) codes (Supplementary Table 1). ASCVD consisted of acute coronary syndrome, other coronary artery disease, ischemic stroke/transient ischemic attack (TIA), and peripheral artery disease. We accepted any record of ASCVD and did not restrict to primary diagnoses. Previous validation studies have shown a 95% positive predictive value (PPV) for having a primary diagnosis of acute coronary syndrome [16, 17], and 98% PPV for stroke/TIA [17]. We are unaware of any validation of peripheral artery disease in Sweden, but a study from Denmark with a similar healthcare system demonstrated a PPV of 69% [18]. ASCVD diagnoses and other comorbidities were identified from the Swedish National Patient Register. Additional demographic and socioeconomic characteristics were obtained from Statistics Sweden.

Among the ESPRESSO cohort, the Index date was defined as 30 days after the first diagnosis of ASCVD and after July 1, 2006. Using unique personal identity numbers, which are assigned to all residents in Sweden [19], we linked all study participants to the Patient Register, Cause of Death Register, and Cancer Register. The Cause of Death Register is effectively complete for all individuals living in Sweden. This register does not include deaths of Swedish expatriates before 2012; however, we censored for emigration prior to our analysis [20]. The Swedish Cancer Register has an estimated data completeness of >96% [21]. Participants were also linked to the Total Population Register (TPR), which contains information on all births and death in Sweden and is estimated to cover 99.5% of all emigrations from Sweden [13]. Family history of CRC was established by identifying family members through the Total Population Register and sequentially linking those relatives to the Swedish Cancer Register.

### Exclusion criteria

**Fig. 1:**
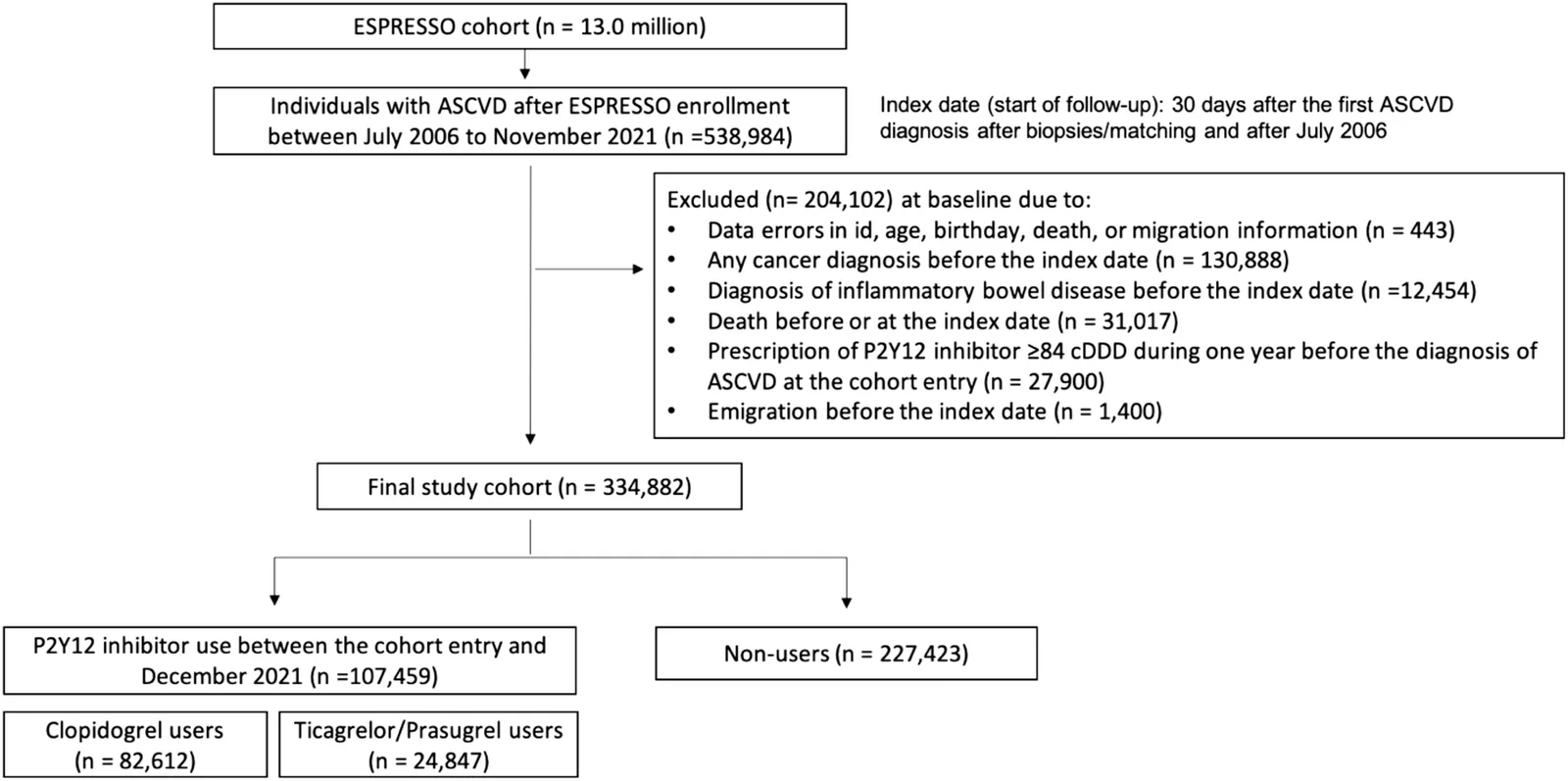
Abbreviations: ASCVD, atherosclerotic cardiovascular disease; cDDD, consecutive defined daily dose

We excluded individuals with a diagnosis of cancer or inflammatory bowel disease prior to cohort entry (Supplementary Table 2). To ensure a new-user design, we excluded individuals with ≥84 cumulative defined daily doses (cDDDs) of P2Y12 inhibitor during the year prior to first diagnosis of ASCVD. We additionally excluded individuals who emigrated prior to cohort entry, given these individuals would be lost to follow-up and not accounted for in our analysis. (Fig. 1).

### Exposure

P2Y12 inhibitor use was a time-varying exposure reflecting the cDDD at each new prescription of P2Y12 inhibitor (clopidogrel, ticagrelor, or prasugrel; recorded by the Anatomical Therapeutic Chemical [ATC] code B01AC04, B01AC24, or B01AC22, respectively). P2Y12 inhibitor users were defined as individuals with prescription of at least 84 cDDD or more of an eligible P2Y12 inhibitor between cohort entry and December 2021. 84 cDDDs represents approximately 12 weeks of treatment and one standard prescription fill. Individuals with <84 cDDDs of P2Y12 inhibitor were not considered to be users. This was chosen as a minimum threshold to represent sustained P2Y12 inhibitor use. For our analysis, individuals were categorized by duration of P2Y12 inhibitor use: 84 to 167 cDDDs (12 weeks to 23 weeks and 6 days), 168 to 335 cDDDs (24 weeks to 47 weeks and 6 days), 336 to 671 cDDDs (48 weeks to 95 weeks and 6 days), ≥ 672 cDDDs ( ≥ 96 weeks). Individuals contributed person-time to each exposure category only after reaching that level of cumulative exposure.

### Outcomes

End of follow-up was defined as CRC diagnosis, cancer, death, emigration, or December 31^st^ 2021, whichever came first. Our primary outcome was incident CRC. Secondary outcomes included CRC mortality, all cancer-related mortality, and all-cause mortality (Supplementary Table 2). We also assessed the risk of metastatic CRC, which was defined as M stage ≥1, regardless of T or N stage identified from the Cancer Register, and non-metastatic CRC.

### Statistical analysis

We used Cox proportional hazards regression models to estimate adjusted hazard ratios (HRs) with 95% confidence intervals (CIs). The proportional hazards assumption was evaluated using Schoenfeld residuals. All individuals contributed person time from the time of their diagnosis of ASCVD. Follow-up began at the time of ASCVD diagnosis. Individuals contributed unexposed person-time until first P2Y12 inhibitor prescription or were censored; upon receiving a P2Y12 inhibitor prescription, when they began accruing person-time to the P2Y12 exposed group. Outcomes occurring before initiation of a P2Y12 inhibitor were attributed to the unexposed person-time. Doing this avoided misclassification of pre-exposure person-time as exposed person-time and thereby addressed potential immortal time bias. We adjusted for age, sex, country of birth (Nordic vs. non-Nordic), total number of clinic visits in the preceding year up until cohort entry, number of hospitalizations in the preceding year, income level according to quantile, educational attainment ( ≤ 9 years, 10–12 years, ≥13 years, missing), types of primary ASCVD, the year of CVD diagnosis (start of follow-up), and a Charlson comorbidity index score. The Charlson comorbidity index score is a weighted sum of a patient’s common comorbid conditions that is used to predict a patients’ morbidity and mortality, and has been adapted to a Swedish setting [22] (Supplementary Table 3). Data on income and education were retrieved from the nationwide LISA database [23]. Income data included individual gross earnings, capital gains, and allowances, such as social welfare. Data on education had an estimated accuracy of 85% [23]. We further adjusted for family history of CRC by identifying study individuals with a first degree relative listed in the Swedish Cancer Register. Finally, we adjusted for regular aspirin use as a dichotomous, non–time-varying covariate defined as ≥84 cDDDs of aspirin (ATC code B01AC06) during the year prior to the start of follow-up. We adjusted for aspirin use at baseline, as modeling aspirin as a time-varying exposure would have required accounting for changes between single- and dual-antiplatelet therapy over follow-up. Missing values for education and family disposable income were included as separate categories. Other covariates were derived from nationwide registry data and had complete information.

We estimated adjusted HRs for primary and secondary outcomes relative to use of any P2Y12 inhibitor (i.e., > = 84 cDDD vs. <84 cDDD), by cDDD category, and according to each individual drug (clopidogrel, ticagrelor, or prasugrel). Based on these results, we subsequently performed a secondary analysis examining the impact of P2Y12 inhibitor use on survival after CRC diagnosis. End of follow-up was defined as death from any cause, emigration, or December 31^st^ 2021, whichever came first. Deaths from causes other than CRC were treated as censoring events for cause-specific hazard ratios. We used log rank test for trend to determine the linear trend between cDDD category of P2Y12 inhibitors and the outcomes. To study new P2Y12 inhibitor use after CRC diagnosis, we excluded individuals with ≥84 cDDDs of P2Y12 inhibitor one year prior to CRC diagnosis. We used the same aforementioned covariates for our Cox proportional hazards regression models and additionally adjusted for tumor stage at diagnosis.

We performed stratified analyses according to sex, age (< 70/ ≥ 70 years), regular aspirin use (yes/no), primary ASCVD diagnosis (acute coronary syndrome/other ASCVD), year of diagnosis (2006-2010/2011-2021), family history of cancer (yes/no), and entry into the ESPRESSO cohort (biopsy/matched control).

All analyses were performed using Stata BE version 17.0. (StataCorp LP, College Station, TX). This study was designed and reported in accordance with the STROBE (Strengthening the Reporting of Observational Studies in Epidemiology) guidelines to ensure transparency and completeness in reporting observational research [24]. This study was approved by the Stockholm Ethics Review Board. Due to the register-based nature of the study informed consent was waived [25].

## Results

We studied 107,459 P2Y12 inhibitor users (82,612 clopidogrel users and 24,847 ticagrelor/prasugrel users) and 227,423 non-users. During a median follow-up of 5.1 years (interquartile range 2.1–9.0), we identified 4,015 incident CRC cases and 2,610 CRC-related deaths. Baseline characteristics according to P2Y12 inhibitor use are shown in Table 1. P2Y12 inhibitor users were younger, more likely to be male, and more likely to have a history of acute coronary syndrome and to be regular aspirin use compared to non-users (Table 1). In contrast, non-users were more likely to have a history of peripheral artery disease and other coronary artery disease (Table 1).

**Table 1:** Baseline characteristics of the study population at the index date

Characteristics	Overall (n = 334882)	P2Y12 inhibitor users ^a^		Non-users ^a^ (n = 227423)
		Clopidogrel (n = 82612)	Ticagrelor/Prasugrel (n = 24847)	
ASCVD at cohort entry, n (%)				
Acute coronary syndrome	71963 (21.5%)	28277 (34.2%)	20307 (81.7%)	23379 (10.3%)
Other coronary artery disease	108314 (32.3%)	16126 (19.5%)	3361 (13.5%)	88827 (39.1%)
Ischemic stroke/TIA	124381 (37.1%)	33680 (40.8%)	870 (3.5%)	89831 (39.5%)
Peripheral arterial disease	30224 (9.0%)	4529 (5.5%)	309 (1.2%)	25386 (11.2%)
ASCVD at first P2Y12 inhibitor prescription, n (%)				
Acute coronary syndrome	NA	18333 (22.2%)	13169 (53.0%)	7 ( < 1%)
Other coronary artery disease	NA	25946 (31.4%)	11364 (45.7%)	2 ( < 1%)
Ischemic stroke/TIA	NA	33646 (40.7%)	180 (0.7%)	5 ( < 1%)
Peripheral arterial disease	NA	4672 (5.7%)	134 (0.5%)	2 ( < 1%)
Others	NA	15 ( < 1%)	0 (0%)	NA
Age, year, median (IQR)	76 (68–84)	73 (66–80)	70 (63–77)	77 (69–84)
Age category, n (%)				
<60	30692 (9.2%)	8711 (10.5%)	4161 (16.7%)	17820 (7.8%)
60–69	69404 (20.7%)	20925 (25.3%)	7837 (31.5%)	40642 (17.9%)
70–79	112535 (33.6%)	30313 (36.7%)	8737 (35.2%)	73485 (32.3%)
≥80	122251 (36.5%)	22663 (27.4%)	4112 (16.5%)	95476 (42.0%)
Female, %	155396 (46.4%)	34623 (41.9%)	7507 (30.2%)	113266 (49.8%)
Country of birth, n (%)				
Nordic country	308060 (92.0%)	75890 (91.9%)	22146 (89.1%)	210024 (92.3%)
Outside Nordic region	26822 (8.0%)	6722 (8.1%)	2701 (10.9%)	17399 (7.7%)
Level of education, n (%)				
≤9 years	130877 (39.1%)	31954 (38.7%)	7689 (30.9%)	91234 (40.1%)
10–12 years	87231 (26.0%)	23041 (27.9%)	7425 (29.9%)	56765 (25.0%)
≥13 years	85205 (25.4%)	23185 (28.1%)	8090 (32.6%)	53930 (23.7%)
Missing	31569 (9.4%)	4432 (5.4%)	1643 (6.6%)	25494 (11.2%)
Family income, n (%)				
Q1 (lowest income)	73680 (22.0%)	16593 (20.1%)	3006 (12.1%)	54081 (23.8%)
Q2	66356 (19.8%)	17742 (21.5%)	3607 (14.5%)	45007 (19.8%)
Q3	60653 (18.1%)	18181 (22.0%)	4300 (17.3%)	38172 (16.8%)
Q4 (highest income)	55060 (16.4%)	16932 (20.5%)	5531 (22.3%)	32597 (14.3%)
Missing	79133 (23.6%)	13164 (15.9%)	8403 (33.8%)	57566 (25.3%)
Number of clinic visits in the previous year	2 (1–4)	2 (1–4)	2 (1–3)	2 (1–4)
Number of hospitalizations in the previous year	1 (1–2)	1 (1–2)	1 (1–2)	1 (1–2)
Year of cohort entry, n (%)				
2006–2010	134361 (40.1%)	33481 (40.5%)	1786 (7.2%)	99094 (43.6%)
2011–2021	200521 (59.9%)	49131 (59.5%)	23061 (92.8%)	128329 (56.4%)
Charlson comorbidity score, median (IQR)	5 (4–6)	5 (3–6)	4 (3–5)	5 (4–6)
Comorbidities included in the Charlson score, n(%)				
Chronic obstructive pulmonary disease/ asthma	43052 (12.9%)	9364 (11.3%)	2553 (10.3%)	31135 (13.7%)
Myocardial infarction	121463 (36.3%)	34148 (41.3%)	20113 (80.9%)	67202 (29.5%)
Congestive heart failure	66269 (19.8%)	10986 (13.3%)	2958 (11.9%)	52325 (23.0%)
Peripheral vascular disease	50905 (15.2%)	9544 (11.6%)	1412 (5.7%)	39949 (17.6%)
Cerebrovascular disease	152235 (45.5%)	39459 (47.8%)	2116 (8.5%)	110660 (48.7%)
Diabetes without complications	69929 (20.9%)	17273 (20.9%)	5218 (21.0%)	47438 (20.9%)
Diabetes with complications	25112 (7.5%)	6067 (7.3%)	1449 (5.8%)	17596 (7.7%)
Rheumatic disease	19799 (5.9%)	4376 (5.3%)	1178 (4.7%)	14245 (6.3%)
Peptic ulcer disease	20716 (6.2%)	4407 (5.3%)	1036 (4.2%)	15273 (6.7%)
Mild or moderate renal disease	16846 (5.0%)	3204 (3.9%)	747 (3.0%)	12895 (5.7%)
Severe renal disease	8765 (2.6%)	1632 (2.0%)	338 (1.4%)	6795 (3.0%)
Paraplegia and hemiplegia	10932 (3.3%)	2842 (3.4%)	199 (0.8%)	7891 (3.5%)
Dementia	16613 (5.0%)	1760 (2.1%)	263 (1.1%)	14590 (6.4%)
Mild liver disease	7212 (2.2%)	1514 (1.8%)	506 (2.0%)	5192 (2.3%)
Moderate or severe liver disease	5719 (1.7%)	1119 (1.4%)	320 (1.3%)	4280 (1.9%)
AIDS/HIV	255 (0.1%)	82 (0.1%)	28 (0.1%)	145 (0.1%)
Family history of colorectal cancer, n (%)	17199 (5.1%)	4662 (5.6%)	1853 (7.5%)	10684 (4.7%)
Regular aspirin use, n (%) ^b^	231633 (69.2%)	60274 (73.0%)	23487 (94.5%)	147872 (65.0%)

*AIDS* acquired immunodeficiency syndrome, *ASCVD* atherosclerotic cardiovascular disease, *HIV* human immunodeficiency virus, *IQR* interquartile range, *TIA* transient ischemic attack.

^a^All patients were non-users at the start of follow-up. Patients were included in the baseline characteristics at the start of follow up was presented here.

^b^Regular aspirin use was defined as the prescription of aspirin ≥84 cDDD in the year prior to the baseline.

Formal tests of proportionality based on Schoenfeld residuals were statistically significant; however, the magnitude of the correlations between the residuals and time was small (absolute rho <0.10), suggesting that the large sample size allowed detection of small variation in the estimated hazard ratios over time. After adjusting for study covariates, no significant association was observed between P2Y12 inhibitor use and CRC incidence (Table 2). However, P2Y12 inhibitor use was significantly associated with lower CRC mortality. Compared to non-users, the adjusted HRs for CRC mortality was 0.97 (95% CI, 0.81–1.17) for individuals with 84–167 cDDDs, 0.87 (95% CI, 0.71–1.07) for individuals with 168–335 cDDDs 0.73 (95% CI, 0.60–0.89) for individuals with 336–671 cDDDs, and 0.81 (95% CI, 0.68–0.97) for individuals with ≥ 672 cDDDs (*P* for trend <0.001) (Table 2). P2Y12 inhibitor use was not associated with all-cancer mortality (0.97, 95% CI 0.93–1.01) but was associated with lower all-cause mortality (0.85, 95% CI 0.84–0.87) (Table 2). We also performed a sensitivity analysis excluding individuals with a history of aspirin use at the start of follow-up, which did not meaningfully change our results.

**Table 2:** Association of P2Y12 inhibitor use with the risk of CRC and mortality

Event and cumulative doses	No. of cases	Person-years	HR (95% CI)		*P* for trend ^b^
			Unadjusted	Multivariable-adjusted ^a^	
**CRC incidence**					
Non-users	3005	1312978	1 [ref]	1 [ref]	
Overall users	1010	595675	0.81 (0.76–0.87)	1.01 (0.93–1.10)	
84–167 cDDDs (12–24 weeks)	302	137658	0.99 (0.88–1.12)	1.09 (0.96–1.24)	<0.001
168–335 cDDDs (24–48 weeks)	230	123162	0.83 (0.73–0.96)	1.04 (0.90–1.21)	
336–671 cDDDs (48–96 weeks)	283	196085	0.69 (0.61–0.79)	0.96 (0.84–1.10)	
≥ 672 cDDDs ( ≥ 96 weeks)	195	139077	0.76 (0.66–0.88)	0.96 (0.82–1.11)	
**CRC-related mortality**					
Non-users	1558	1323892	1 [ref]	1 [ref]	
Overall users	521	600850	0.73 (0.66–0.80)	0.84 (0.75–0.94)	
84–167 cDDDs (12–24 weeks)	142	138858	0.87 (0.73–1.03)	0.97 (0.81–1.17)	<0.001
168–335 cDDDs (24–48 weeks)	108	124130	0.76 (0.62–0.92)	0.87 (0.71–1.07)	
336–671 cDDDs (48–96 weeks)	135	197643	0.58 (0.48–0.69)	0.73 (0.60–0.89)	
≥ 672 cDDDs ( ≥ 96 weeks)	136	140528	0.77 (0.64–0.92)	0.81 (0.68–0.97)	
**Cancer-related mortality**					
Non-users	13653	1323892	1 [ref]	1 [ref]	
Overall users	4988	600850	0.82 (0.79–0.84)	0.97 (0.93–1.01)	
84–167 cDDDs (12–24 weeks)	1225	138858	0.86 (0.81–0.91)	0.98 (0.92–1.04)	<0.001
168–335 cDDDs (24–48 weeks)	998	124130	0.81 (0.76–0.87)	0.96 (0.89–1.02)	
336–671 cDDDs (48–96 weeks)	1409	197643	0.72 (0.68–0.76)	0.91 (0.86–0.97)	
≥ 672 cDDDs ( ≥ 96 weeks)	1356	140528	0.92 (0.87–0.97)	1.02 (0.96–1.08)	
**All-cause mortality**					
Non-users	124361	1323892	1 [ref]	1 [ref]	
Overall users	33794	600850	0.63 (0.62–0.64)	0.85 (0.84–0.87)	
84–167 cDDDs (12–24 weeks)	8768	138858	0.68 (0.67–0.70)	0.86 (0.83–0.88)	<0.001
168–335 cDDDs (24–48 weeks)	6750	124130	0.61 (0.60–0.63)	0.83 (0.81–0.86)	
336–671 cDDDs (48–96 weeks)	8385	197643	0.49 (0.48–0.50)	0.77 (0.75–0.79)	
≥ 672 cDDDs ( ≥ 96 weeks)	9905	140528	0.79 (0.77–0.80)	0.94 (0.92–0.96)	

*ASCVD* atherosclerotic cardiovascular disease, *cDDD* consecutive defined daily dose, *CI* confidence interval, *CRC* colorectal cancer, *HR* hazard ratio.

^a^Multivariable model was adjusted for age, sex, country of birth, previous clinic visits, previous hospitalization, income levels, education background, Charlson comorbidity index score, family history of CRC, primary ASCVD, regular aspirin use, and the year of index date.

^b^*P* for trend was estimated by the log-rank test for trend.

All P2Y12 inhibitors were associated with lower CRC mortality. The majority of patients were prescribed clopidogrel. Given the relatively limited number of individuals who were prescribed ticagrelor or prasugrel, we combined users of these agents into one category Compared to nonusers, patients prescribed clopidogrel experienced an adjusted HR for CRC mortality of 0.86 (95% CI, 0.77–0.97) and patients prescribed ticagrelor or prasugrel experienced an adjusted HR for CRC mortality of 0.63 (95%, 0.48–0.83) (Table 3). No agent was individually associated with CRC incidence.

**Table 3:** Association of clopidogrel, non-clopidogrel P2Y12 inhibitor use with the risk of CRC and mortality

Event and drug	No. of cases	Person-years	HR (95% CI)	
			Unadjusted	Multivariable-adjusted ^a^
**CRC incidence**				
Non-users	3005	1312978	1 [ref]	1 [ref]
Clopidogrel	878	485235	0.89 (0.83–0.96)	1.02 (0.94–1.11)
Ticagrelor/Prasugrel	132	110440	0.52 (0.43–0.61)	0.96 (0.79–1.16)
**CRC-related mortality**				
Non-users	1558	1323892	1 [ref]	1 [ref]
Clopidogrel	462	489851	0.78 (0.71–0.87)	0.86 (0.77–0.97)
Ticagrelor/Prasugrel	59	110999	0.47 (0.36–0.61)	0.63 (0.48–0.83)
**Cancer-related mortality**				
Non-users	13653	1323892	1 [ref]	1 [ref]
Clopidogrel	4341	489851	0.87 (0.84–0.90)	0.99 (0.95–1.02)
Ticagrelor/Prasugrel	647	110999	0.60 (0.55–0.65)	0.83 (0.76–0.90)
**All-cause mortality**				
Non-users	124361	1323892	1 [ref]	1 [ref]
Clopidogrel	30334	489851	0.69 (0.68–0.70)	0.87 (0.85–0.89)
Ticagrelor/Prasugrel	3460	110999	0.35 (0.34–0.37)	0.70 (0.68–0.73)

^a^Multivariable model was adjusted for age, sex, country of birth, previous clinic visits, previous hospitalization, income levels, education background, Charlson comorbidity index score, family history of CRC, primary ASCVD, and regular aspirin use, and the year of index date).

Abbreviations: ASCVD, atherosclerotic cardiovascular disease CI, confidence interval; CRC, colorectal cancer; HR, hazard ratio

To further understand how P2Y12 inhibitor use might contribute to lower CRC mortality, we considered whether this may be related to the stage of CRC at time of diagnosis. No association, however, was observed in sub-analyses of nonmetastatic and metastatic CRC (Supplementary table 4). We additionally stratified according to sex, age, regular aspirin use, and primary CVD diagnosis, and found no significant interactions (Supplementary table 5). To remove the possibility of occult CRC at cohort entry, we performed a secondary analysis excluding individuals with a CRC diagnosis within 2 years after index year. The association between P2Y12 inhibitor use and CRC mortality remained largely unchanged after exclusion (Supplementary table 6).

Given the association between P2Y12 inhibitors and CRC mortality in the absence of an association with CRC incidence, we considered whether initiation of a P2Y12 inhibitor after incident CRC was associated with lower CRC mortality. We identified 2,948 individuals with incident CRC and without prior P2Y12 inhibitor use with a median follow-up of 2.52 year. Among them, 292 individuals initiated P2Y12 inhibitor after diagnosis. Individuals who initiated a P2Y12 inhibitor after diagnosis had an adjusted HR for CRC mortality of 0.48 (95% CI, 0.35–0.65) compared to non-users, and the association was not materially altered by adjustment for CRC stage at diagnosis (Table 4).

**Table 4:** Association of initiation of P2Y12 inhibitor after CRC diagnosis with the risk of mortality among patients with incident CRC (n = 2,948)

Events	No. of deaths	Person-years	HR (95% CI)	
			Unadjusted	Multivariable-Adjusted ^a^
**CRC-related mortality**				
Non-users	783	10066	1 [ref]	1 [ref]
P2Y12 inhibitor users	27	1081	0.29 (0.20–0.43)	0.42 (0.29–0.60)
**Cancer-related mortality**				
Non-users	952	10066	1 [ref]	1 [ref]
P2Y12 inhibitor users	51	1081	0.46 (0.95–0.60)	0.62 (0.47–0.82)
**All-cause mortality**				
Non-users	1861	10066	1 [ref]	1 [ref]
P2Y12 inhibitor users	164	1081	0.79 (0.68–0.91)	0.93 (0.79–1.11)

^a^Multivariable model was adjusted for age, sex, country of birth, previous clinic visits, previous hospitalization, income levels, education background, Charlson comorbidity index score, family history of CRC, primary ASCVD, and the year of CRC diagnosis, aspirin use prior to CRC diagnosis, and CRC stage (I, II, III, IV, and missing) at diagnosis.

Abbreviations: ASCVD, atherosclerotic cardiovascular disease; CI, confidence interval; CRC, colorectal cancer; HR, hazard ratio

## Discussion

In this nationwide study of Swedish individuals diagnosed with ASCVD, P2Y12 inhibitor use was not associated with CRC incidence but significantly associated with lower CRC mortality. A protective association on CRC mortality was seen among all P2Y12 inhibitors, including clopidogrel, ticagrelor, and prasugrel. Initiation of P2Y12 use in individuals after diagnosis of CRC was linked to more than a 50% decrease in CRC mortality.

There is substantial epidemiologic research showing a preventive effect of aspirin on the development of CRC [2–5]. Since it was first observed in a population-based case-control study in 1988 [26], numerous cohort and case–control studies, secondary analyses of RCTs of aspirin on cardiovascular prevention, and long-term follow-up of the Women’s Health Study [27], have reported an inverse association between aspirin use and risk of CRC [2, 3]. A causal association of aspirin with CRC neoplasia has been established through randomized trials of aspirin among individuals with a history of colorectal adenoma or cancer that show a reduction in recurrent adenoma and individuals with Lynch syndrome that show a reduction in long-term risk of CRC [5, 28]. Finally, several observational studies have shown that aspirin after the diagnosis of CRC is associated with improved CRC-specific survival [29, 30], although this has not been observed universally [31].

While the exact mechanisms by which aspirin exerts a preventive effect remain uncertain, several interrelated biological pathways have been proposed. These include inhibition of prostaglandin synthesis, interference of WNT–β-catenin signaling, and modulation of the host immune system. Moreover, there may be other distinct biological pathways by which aspirin influences disease progression and metastasis [8]. For example, aspirin use has been shown to have a larger impact on the progression of PIK3CA-mutant CRC compared to PIK3CA wild-type CRC but no differential impact on incident CRC [32, 33].

The expression of PTGS-2 on epithelia of the large intestine is ultimately thought to be key to the development of CRC [34]. However, low-dose aspirin inhibits platelet-PTGS-1 and does not appear to be sufficiently able to directly inhibit PTGS-2 in colorectal epithelium [35]. For this reason, the anti-cancer effect of aspirin has been hypothesized to be mediated, in part, by inhibition of platelet-derived COX-1. It remains unknown if antiplatelet agents with different mechanisms of inhibition have preventive effects similar to aspirin. It is also unclear if the mechanisms that underlie reduction in risk of incident colorectal neoplasia are the same as those associated with improved survival after diagnosis.

Our results add to a growing body of evidence that suggests inhibition of platelets improves survival in patients diagnosed with CRC and offer new evidence that the mechanisms by which antiplatelet medications influence the development of CRC may differ from those that influence tumor progression and metastasis. We show a duration-dependent decreased mortality risk among individuals prescribed P2Y12 inhibitors, which persisted after adjusting for aspirin (Table 2). In addition, this effect remained largely unchanged after exclusion of new CRC cases within two years of cohort entry (Supplementary Table 6). Interestingly, we did not observe an association between P2Y12 inhibitor use and CRC incidence (Table 2). This differs from Rodríguez-Miguel et al (2019), who previously found clopidogrel monotherapy was associated with a significantly reduced incidence of CRC [12]. Their findings, however, were significant only after adjusting for other risk factors and the duration of use yielded inconsistent results [36]. Still, a few other studies have similarly observed an association between P2Y12 inhibitor use and lower CRC incidence [37, 38].

We found that among individuals diagnosed with CRC, those who initiated a P2Y12 inhibitor after diagnosis of CRC had more than a 50% reduced CRC-related mortality risk compared to non-users (Table 4). The association between P2Y12 inhibitor use and mortality in our study appeared strongest for survival after CRC diagnosis. While this post-diagnosis secondary analysis provides a potential explanation for the reduced CRC-related mortality observed in our primary analysis, we cannot completely exclude healthy-user bias among patients who initiated a P2Y12 inhibitor after diagnosis. Similarly, a mechanism accounting for this finding remains unknown, but some *in vitro* studies suggest inhibition of platelets via the P2Y12 receptor could influence metastatic spread and survival [39–41]. Ticagrelor has been shown *in vitro* to prevent the down-regulation of E-cadherin and inhibit the production of thromboxane B2 and prostaglandin E2 in CRC cells cocultured with platelets [39]. Both the down-regulation of E-cadherin and the production of thromboxane B2 and prostaglandin E2 are considered essential steps in epithelial-mesenchymal transition (EMT), a process in which epithelial cells develop mesenchymal characteristics that allow for invasion and metastasis [40]. Furthermore, in the TICONC (Ticagrelor-Oncology) Study, ticagrelor was shown *in vitro* to prevent large CRC-cell induced platelet aggregates and CRC cell−endothelial cell adhesion [41]. Tumor cell-induced platelet aggregation (TCIPA) is thought to promote metastatic spread by protecting circulating tumor cells from shear force and concealing tumor cells from immune surveillance [40]. The involvement of platelets in EMT and TCIPA—events essential for metastatic spread—may explain how P2Y12 inhibitor use was associated with lower CRC mortality and not incidence CRC. Recently, researchers similarly found that inhibition of COX-1, including aspirin, enhances T-cell immunity to cancer metastases by reducing platelet-derived thromboxane A2 (TXA2) [42]. These studies provides a possible mechanism by which platelet inhibition could influence metastatic spread and survival.

Clopidogrel was the most prescribed P2Y12 inhibitor in this study and was associated with a 15% decreased CRC-related mortality risk compared to nonusers (Table 3). Due to limited numbers of users of non-clopidogrel agents, we combined ticagrelor and prasugrel in our sub-analysis. Users of either ticagrelor or prasugrel had an almost 40% reduced CRC-mortality risk compared to non-users (Table 3). This difference in mortality risk is unclear. Clopidogrel is a prodrug metabolized to its active metabolite in the liver via cytochrome P450 (CYP) 2C19 whereas prasugrel is activated by the GI tract and ticagrelor does not require metabolic activation. As a result, prasugrel and ticagrelor have little variability in efficacy, while the activity of clopidogrel is potentially susceptible to inhibition of CYP2C19 by other medications [43]. It is unclear if differences in metabolism, differences in action, or unmeasured sources of confounding explain mortality differences observed in our study.

This study has several strengths. This was a large, population-based cohort study of 334,882 individuals with a median follow-up of more than 5 years and prospectively updated data on exposure. While the ESPRESSO cohort was formed around individuals with GI histopathology, stratified analyses found no significant difference in CRC incidence and CRC mortality according to histopathology status. Data on both cohort formation, exposures and outcomes were retrieved from validated nationwide registers. For instance, the Swedish Patient Register has a positive predictive value of 85–95% for most diagnoses [17], the Prescribed Drug Register has missing identity information on less than 0.3% of all prescriptions [15], both the Cause of Death Register and the Cancer Register have high coverage, with some 99% of registered cancers being morphologically verified [20, 21]. The data collated among these registers allowed us to adjust for a large number of covariates. This included comorbidities, number of hospital admissions and clinic visits, family history of CRC, and income and education from the LISA database [23]. Individual-level data on dispensed medications enabled us to separately examine P2Y12 inhibitors on CRC incidence with and without aspirin at the start of follow-up.

### Limitations of the study

This study also had several limitations. Given participants were identified through having undergone gastrointestinal biopsy, there is potential for selection bias related to the indication for biopsy, although each participant was matched with up to five reference individuals from the general population. There is potential for survival bias because individuals with greater cumulative P2Y12 inhibitor exposure must remain alive long enough to accrue greater exposure. To address this, we performed a 12-month landmark analysis in which exposure status was defined within the first year after ASCVD diagnosis and follow-up began thereafter. Results from the landmark analysis were generally consistent with the primary analysis (Supplementary Table 7). Secondary analyses were limited by smaller number of exposed individuals and events. For instance, there were substantially fewer prescriptions for ticagrelor and prasugrel compared to clopidogrel and were therefore combined to provide more precise estimates. Similarly, the mortality restricted analysis had substantially less exposed follow-up time, with only 1,081 person-years among P2Y12 inhibitor users, although the observed association remained significant. We additionally recognize the potential for confounding by indication, as patient characteristics that determine who gets P2Y12 therapy may themselves be associated with the outcome, which can be particularly true for CRC-related mortality. We sought to reduce this concern by adjusting for potential confounders, including baseline cardiovascular disease and comorbidity burden. Similarly, adjusting for prior clinic visits and hospitalizations may not fully account for differences in healthcare utilization. Individuals who initiate P2Y12 inhibitor therapy after a CRC diagnosis may represent a selected subgroup with better underlying health or greater ability to tolerate treatment. To address differences in prognosis, we adjusted for CRC stage and Charlson Comorbidity Index in our models. However, we acknowledge that these factors may not fully capture all aspects of health status and residual healthy user bias cannot be completely excluded. Given we restricted our analysis to people diagnosed with ASCVD, competing cardiovascular events may influence the observed risks of CRC incidence and CRC-related mortality. However, P2Y12 inhibitor therapy should primarily reduce the competing risk of cardiovascular mortality and by consequence preserve an individual’s at-risk status for CRC-related outcomes. 84 cDDDs was chosen as a minimum exposure threshold given medication adherence cannot be confirmed by dispensing data, but a trade-off of this approach is potential exposure misclassification. Finally, we cannot exclude unmeasured residual confounding. The Swedish national healthcare registers lack data on body mass index (BMI) and cigarette smoking, both of which have been shown to be independently associated with CRC incidence and mortality [44]. The Charlson comorbidity index score includes chronic pulmonary disease [22], which likely accounts for smoking-related respiratory conditions; however, we cannot exclude the possibility of residual or unmeasured confounding. Although we adjusted for aspirin use at cohort entry, changes in aspirin use during follow-up were not captured, and this may result in residual confounding. Our study population was limited to individuals with a diagnosis of ASCVD and our results may not be generalizable to all individuals. In addition, approximately 92% of our study population were of Nordic origin and less than 10% of individuals were <60 years of age, both of which may further limit external validity. Finally, Sweden did not have an organized CRC screening program during most of our follow-up period. The relative risks of CRC incidence and mortality in our study may differ from those where CRC screening is prevalent [45].

## Conclusions

In conclusion, P2Y12 inhibitor use was not associated with CRC incidence but associated with improved CRC mortality among individuals diagnosed with ASCVD in the ESPRESSO cohort. Mortality benefits appeared greatest for survival after CRC diagnosis. These findings raise the possibility that the mechanisms underlying the association between aspirin and reduced CRC incidence may differ from those underlying the association between aspirin and CRC survival. If confirmed in additional cohorts, including those without ASCVD, including those without ASCVD, inhibition of platelets via the P2Y12 receptor could be explored as a potential adjuvant treatment for patients after a diagnosis of CRC.

## Supplementary information

Supplementary Information

## Data Availability

Other researchers can apply for our data through the different Swedish pathology departments, and through the Swedish National Board of Health and Welfare.

## Abbreviations

CRC: Colorectal cancer
ESPRESSO: Epidemiology Strengthened by histoPathology Reports in Sweden
PTGS: Prostaglandin-endoperoxide synthase
USPSTF: United States Preventive Services Taskfor
