# Supplementary material for: Platelet P2Y12 inhibitor use and risk of colorectal cancer incidence and colorectal cancer-related mortality: a Swedish registry-based cohort study
Source: BMC Gastroenterol. 2026 Oct 12;26:641. doi: 10.1186/s12876-026-05422-z (PMC13660269; doi:10.1186/s12876-026-05422-z)
Supplement: Supplementary file 1 [file 12876_2026_5422_MOESM1_ESM.docx]

| **Supplementary Table 1. Diagnosis codes for the definition of atherosclerotic cardiovascular diseases** | |
| --- | --- |
| **Diseases** | **ICD-10** |
| Acute coronary syndrome (myocardial infarction/ unstable angina) | I21, I22, I20.0 |
| Other coronary artery disease | I20.1-I20.9, I25.1, I25.2, I25.5, I25.6 |
| Ischemic stroke/transient ischemic attack | I63, I64, G45 |
| Peripheral arterial disease | I70, I73.9 |
| Abbreviation: ICD-10, the 10^th^ revision of the International Classification of Diseases | |

| **Supplementary Table 2. Diagnosis codes for exclusion and outcome evaluation** | | |
| --- | --- | --- |
| **Condition** | **Source** | **ICD codes** |
| *Exclusion* | | |
| Any cancer (other than non-melanoma skin cancer) | Cancer Register | ICD-7:140-190, 192-208  ICD-10: C00-43, C45-97 |
| Inflammatory bowel disease | Patient Register | ICD-7: 572.00, 572.09, 572.20, 572.21  ICD-8: 563, 563.10, 563.99, 569.02  ICD-9: 555, 556  ICD-10: K50, K51, K52.3 |
| *Outcomes* | | |
| Colorectal cancer | Cancer Register | ICD-7: 153-154  ICD-10: C18-20 |
| Colorectal cancer-related mortality | Cause of Death Register | ICD-10: C18-20 |
| Abbreviations: ICD-7, the 7^th^ revision of the International Classification of Diseases; ICD-8, the 8^th^ revision of the International Classification of Diseases; ICD-9, the 9^th^ revision of the International Classification of Diseases; ICD-10, the 10^th^ revision of the International Classification of Diseases | | |

| **Supplementary Table 3. ICD-10 codes for the Charlson comorbidity index score** | |
| --- | --- |
| **Conditions** | **ICD-10** |
| Chronic obstructive pulmonary disease/ asthma | J44.0, J44.1, J44.8, J44.9, J45.0, J45.1, J45.8, J45.9, J46 |
| Myocardial infarction | I21.0, I21.1, I21.2, I21.3, I21.4, I21.4A, I21.4B, I21.4W, I21.4X, I21.9, I22.0, I22.1, I22.8, I22.9, I25.2 |
| Cerebrovascular disease | G45.0, G45.1, G45.2, G45.3, G45.4, G45.8, G45.9, G46.0, G46.1, G46.2, G46.3, G46.4, G46.5, G46.6, G46.7, G46.8, H34.0, I60.0, I60.1, I60.2, I60.3, I60.4, I60.5, I60.6, I60.7, I60.8, I60.9, I61.0, I61.1, I61.2, I61.3, I61.4, I61.5, I61.6, I61.8, I61.9, I62.0, I62.1, I62.9, I63.0, I63.1, I63.2, I63.3, I63.4, I63.5, I63.6, I63.8, I63.9, I64.9, I65.0, I65.1, I65.2, I65.3, I65.8, I65.9, I66.0, I66.1, I66.2, I66.3, I66.4, I66.8, I66.9, I67.0, I67.1, I67.2, I67.3, I67.4, I67.5, I67.6, I67.7, I67.8, I67.9, I68.0, I68.1, I68.2, I68.8, I69.0, I69.1, I69.2, I69.3, I69.4, I69.8 |
| Peripheral vascular disease | I70.0, I70.1, I70.2, I70.8, I70.9, I71.0, I71.1, I71.2, I71.3, I71.4, I71.5, I71.6, I71.8, I71.9, I73.1, I73.8, I73.9, I77.1, I79.0, I79.2, K55.1, K55.8, K55.9, Z95.8, Z95.9 |
| Congestive heart failure | I11.0, I13.0, I13.2, I25.5, I42.0, I42.5, I42.6, I42.7, I42.8, I42.9, I43, I50 |
| Diabetes without complications | E08.0, E08.1, E08.8, E08.9, E09.0, E09.1, E09.8, E09.9, E10.1, E10.8, E10.9, E11.0, E11.1, E11.8, E11.9, E13.0, E13.1, E13.8, E13.9 |
| Diabetes with complications | E08.2, E08.3, E08.4, E08.5, E08.6, E09.2, E09.3, E09.4, E09.5, E09.6, E10.2, E10.3, E10.4, E10.5, E10.6, E11.2, E11.3, E11.4, E11.5, E11.6, E13.2, E13.3, E13.4, E13.5, E13.6 |
| Connective tissue disease-rheumatic disease | M05, M06, M31.5, M32, M33, M34, M35.1, M35.3, M36.0 |
| Peptic ulcer disease | K25, K26, K27, K28 |
| Mild or moderate renal disease | I12.9, I13.0, I13.10, N03, N05, N18.1, N18.2, N18.3, N18.4, N18.9, Z94.0 |
| Severe renal disease | I12.0, I13.11, I13.2, N18.5, N18.6, N19, N25.0, Z49, Z99.2 |
| Paraplegia and hemiplegia | G04.1, G11.4, G80.0, G80.1, G80.2, G81, G82, G83 |
| Dementia | F00.0, F00.1, F00.2, F00.9, F01.0, F01.1, F01.2, F01.3, F01.8, F01.9, F02.0, F02.1, F02.2, F02.3, F02.4, F02.8, F03, F05.1, G30.0, G30.1, G30.8, G30.9, G31.1 |
| Mild liver disease | B18, K70.0, K70.1, K70.2, K70.3, K70.9, K71.3, K71.4, K71.5, K71.7, K73, K74, K76.0, K76.2, K76.3, K76.4, K76.8, K76.9, Z94.4 |
| Moderate or severe liver disease | K71, K721, K73, K74, K75, K76, R18 |
| AIDS/HIV | B20, B21, B22, B23, B24 |
| Solid tumor, leukemia, and lymphoma were not included, as all patients with malignancy were excluded at the baseline.  Abbreviation: AIDS, acquired immunodeficiency syndrome; HIV, human immunodeficiency virus; ICD-10, the 10^th^ revision of the International Classification of Diseases | |

| **Supplementary Table 4. Association of P2Y12 inhibitor use with the risk of non-metastatic and metastatic CRC** | | | | |
| --- | --- | --- | --- | --- |
| **Event and cumulative doses** | **No. of cases** | **Person-years** | **HR (95% CI)** | |
|  |  |  | **Unadjusted** | **MV-adjusted ^a^** |
| **Non-metastatic CRC** |  |  |  |  |
| Non-users | 2626 | 1313459 | 1 [ref] | 1 [ref] |
| Overall users | 892 | 595846 | 0.83 (0.77-0.89) | 1.03 (0.95-1.13) |
| 84 – 167 cDDDs  (12-24 weeks) | 271 | 137706 | 1.02 (0.90-1.16) | 1.13 (0.98-1.29) |
| 168-335 cDDDs  (24-48 weeks) | 211 | 123186 | 0.88 (0.76-1.01) | 1.11 (0.95-1.29) |
| 336-671 cDDDs  (48-96 weeks) | 244 | 195849 | 0.69 (0.60-0.79) | 0.96 (0.83-1.11) |
| ≥ 672 cDDDs  (≥96 weeks) | 166 | 139110 | 0.75 (0.64-0.88) | 0.95 (0.80-1.12) |
| **Metastatic (Stage IV) CRC** |  |  |  |  |
| Non-users | 379 | 1323411 | 1 [ref] | 1 [ref] |
| Overall users | 118 | 600679 | 0.72 (0.59-0.89) | 0.88 (0.69-1.12) |
| 84 – 167 cDDDs  (12-24 weeks) | 31 | 138810 | 0.81 (0.56-1.17) | 0.85 (0.58-1.26) |
| 168-335 cDDDs  (24-48 weeks) | 19 | 124107 | 0.54 (0.34-0.86) | 0.65 (0.40-1.05) |
| 336-671 cDDDs  (48-96 weeks) | 39 | 197273 | 0.72 (0.52-1.01) | 0.96 (0.66-1.38) |
| ≥ 672 cDDDs  (≥ 96 weeks) | 29 | 140495 | 0.84 (0.57-1.23) | 1.01 (0.68-1.50) |
| ^a^ Multivariable model was adjusted for age, sex, country of birth, previous clinic visits, previous hospitalization, income levels, education background, Charlson comorbidity index score, family history of CRC, primary ASCVD, regular aspirin use, and the year of index date.  Abbreviations: ASCVD, atherosclerotic cardiovascular disease; cDDD, consecutive defined daily dose; CI, confidence interval; CRC, colorectal cancer; HR, hazard ratio | | | | |

| **Supplementary Table 5. Stratified associations between P2Y12 inhibitor use and incident CRC and CRC-related mortality** | | | | | | |
| --- | --- | --- | --- | --- | --- | --- |
|  | **Incident CRC** | | | **CRC-related mortality** | | |
| **Subgroups** | **No. of cases** | **Person-year** | **Adjusted HR (95% CI) ^a^** | **No. of cases** | **Person-year** | **Adjusted HR (95% CI) ^a^** |
| **By sex** |  |  |  |  |  |  |
| Male | 2466 | 1050197 | 0.98 (0.88-1.09) | 1199 | 1059956 | 0.76 (0.66-0.88) |
| Female | 1549 | 858456 | 1.08 (0.94-1.24) | 880 | 864787 | 0.97 (0.81-1.16) |
| *P* for interaction |  |  | .06 |  |  | .09 |
| **By age, year** |  |  |  |  |  |  |
| <70 | 1046 | 772394 | 1.08 (0.92-1.26) | 375 | 777652 | 0.90 (0.70-1.16) |
| ≥70 | 2969 | 1136259 | 0.98 (0.88-1.08) | 1704 | 1147091 | 0.78 (0.69-0.89) |
| *P* for interaction |  |  | .94 |  |  | .32 |
| **By regular aspirin use** |  |  |  |  |  |  |
| Yes | 2899 | 1402796 | 1.03 (0.93-1.14) | 1438 | 1414742 | 0.81 (0.71-0.93) |
| No | 1116 | 505857 | 1.01 (0.86-1.20) | 641 | 510000 | 0.92 (0.75-1.14) |
| *P* for interaction |  |  | .31 |  |  | .69 |
| **By primary diagnosis** |  |  |  |  |  |  |
| Acute coronary syndrome | 767 | 422375 | 0.95 (0.80-1.12) | 388 | 425513 | 0.74 (0.59-0.92) |
| Other ASCVD | 3248 | 1486278 | 1.05 (0.96-1.16) | 1691 | 1499230 | 0.89 (0.78-1.01) |
| *P* for interaction |  |  | .09 |  |  | .11 |
| **By year of diagnosis** |  |  |  |  |  |  |
| 2006-2010 | 2471 | 1004718 | 0.97 (0.87-1.09) | 1120 | 1015483 | 0.83 (0.70-0.97) |
| 2011-2021 | 1544 | 903935 | 1.08 (0.95-1.23) | 959 | 909259 | 0.88 (0.75-1.04) |
| *P* for interaction |  |  | .43 |  |  | .83 |
| **By family history** |  |  |  |  |  |  |
| Family history of CRC | 242 | 101724 | 0.89 (0.63-1.26) | 106 | 102879 | 0.62 (0.36-1.06) |
| No family history of CRC | 3773 | 1806929 | 1.02 (0.94-1.11) | 1973 | 1821864 | 0.86 (0.76-0.96) |
| *P* for interaction |  |  | .16 |  |  | .20 |
| **By ESPRESSO cohort entry** |  |  |  |  |  |  |
| Biopsy | 1836 | 949939 | 1.11 (0.98-1.26) | 871 | 957562 | 0.86 (0.72-1.02) |
| Matched control | 2179 | 958714 | 0.93 (0.83-1.05) | 1208 | 967180 | 0.83 (0.71-0.96) |
| *P* for interaction |  |  | .36 |  |  | .71 |
| ^a^ Multivariable model was adjusted for age, sex, country of birth, previous clinic visits, previous hospitalization, year of CVD diagnosis (start of follow-up), income levels, education background, Charlson comorbidity index score, family history of CRC, primary ASCVD, regular aspirin use, and the year of index date.  Abbreviations: ASCVD, atherosclerotic cardiovascular disease CI, confidence interval; CRC, colorectal cancer; HR, hazard ratio; PY, person-year | | | | | | |

| **Supplementary Table 6. Risk of incident CRC and CRC-related mortality according to P2Y12 inhibitor use, after excluding CRC diagnosis within <2 years** | | | | |
| --- | --- | --- | --- | --- |
| **Events and cumulative doses** | **No. of cases** | **Person-years** | **HR (95% CI)** | |
|  |  |  | **Unadjusted** | **MV-adjusted ^a^** |
| **CRC incidence** |  |  |  |  |
| Non-users | 1649 | 1311809 | 1 [ref] | 1 [ref] |
| Overall users | 709 | 595445 | 0.86 (0.78-0.94) | 1.08 (0.97-1.19) |
| 84 – 167 cDDDs  (12-24 weeks) | 183 | 137553 | 1.09 (0.94-1.27) | 1.14 (0.97-1.34) |
| 168-335 cDDDs  (24-48 weeks) | 136 | 123081 | 0.95 (0.80-1.13) | 1.13 (0.94-1.37) |
| 336-671 cDDDs  (48-96 weeks) | 204 | 195742 | 0.74 (0.64-0.85) | 1.05 (0.89-1.24) |
| ≥ 672 cDDDs  (≥96 weeks) | 186 | 139075 | 0.78 (0.67-0.90) | 1.02 (0.87-1.19) |
| **CRC-related mortality** |  |  |  |  |
| Non-users | 1207 | 1317289 | 1 [ref] | 1 [ref] |
| Overall users | 438 | 598509 | 0.77 (0.69-0.86) | 0.86 (0.76-0.97) |
| 84 – 167 cDDDs  (12-24 weeks) | 120 | 138276 | 0.92 (0.76-1.11) | 1.02 (0.83-1.24) |
| 168-335 cDDDs  (24-48 weeks) | 83 | 123575 | 0.77 (0.61-0.96) | 0.86 (0.68-1.08) |
| 336-671 cDDDs  (48-96 weeks) | 110 | 196658 | 0.61 (0.50-0.74) | 0.75 (0.60-0.93) |
| ≥ 672 cDDDs  (≥ 96 weeks) | 125 | 140005 | 0.83 (0.69-0.99) | 0.83 (0.69-1.01) |
| ^a^ Multivariable model was adjusted for age, sex, country of birth, previous clinic visits, previous hospitalization, income levels, education background, Charlson comorbidity index score, family history of CRC, primary ASCVD, regular aspirin use, and the year of index date.  Abbreviations: ASCVD, atherosclerotic cardiovascular disease; cDDD, consecutive defined daily dose; CI, confidence interval; CRC, colorectal cancer; HR, hazard ratio | | | | |

| **Supplementary Table 7: Landmark analysis for association of P2Y12 inhibitor use with the risk of CRC and mortality** | | | | | |
| --- | --- | --- | --- | --- | --- |
| **Event and cumulative doses** | **No. of cases** | **Person-years** | **HR (95% CI)** | | ***P* for trend ^b^** |
|  |  |  | **Unadjusted** | **Multivariable-adjusted ^a^** |  |
| **CRC incidence** |  |  |  |  |  |
| Non-users | 1087 | 609660 | 1 [ref] | 1 [ref] |  |
| Overall users | 1907 | 992909 | 1.11 (1.03–1.20) | 1.00 (0.93–1.08) |  |
| 84-167 cDDDs  (12-24 weeks) | 110 | 58555 | 1.07 (0.88–1.31) | 1.03 (0.85–1.25) | 0.77 |
| 168-335 cDDDs  (24-48 weeks) | 594 | 316629 | 1.08 (0.97–1.19) | 0.99 (0.89–1.09) |  |
| 336-671 cDDDs  (48-96 weeks) | 1107 | 567341 | 1.14 (1.05–1.24) | 1.02 (0.93–1.11) |  |
| ≥ 672 cDDDs  (≥96 weeks) | 96 | 50384 | 1.11 (0.90–1.37) | 0.92 (0.74–1.13) |  |
| **CRC-related mortality** |  |  |  |  |  |
| Non-users | 711 | 615204 | 1 [ref] | 1 [ref] |  |
| Overall users | 1057 | 1002956 | 0.90 (0.82–0.99) | 0.90 (0.81–0.99) |  |
| 84-167 cDDDs  (12-24 weeks) | 81 | 59201 | 1.17 (0.93–1.48) | 1.22 (0.97–1.54) | 0.03 |
| 168-335 cDDDs  (24-48 weeks) | 357 | 319831 | 0.96 (0.84–1.09) | 0.95 (0.83–1.08) |  |
| 336-671 cDDDs  (48-96 weeks) | 574 | 573064 | 0.85 (0.76–0.95) | 0.85 (0.76–0.95) |  |
| ≥ 672 cDDDs  (≥96 weeks) | 45 | 50860 | 0.75 (0.56–1.02) | 0.72 (0.54–0.98) |  |
| **Cancer-related mortality** |  |  |  |  |  |
| Non-users | 5949 | 615204 | 1 [ref] | 1 [ref] |  |
| Overall users | 9411 | 1002956 | 0.96 (0.93–0.99) | 0.95 (0.91–0.98) |  |
| 84-167 cDDDs  (12-24 weeks) | 695 | 59201 | 1.20 (1.11–1.30) | 1.23 (1.13–1.33) | <.001 |
| 168-335 cDDDs  (24-48 weeks) | 3138 | 319831 | 1.00 (0.96–1.05) | 0.98 (0.94–1.03) |  |
| 336-671 cDDDs  (48-96 weeks) | 5166 | 573064 | 0.92 (0.88–0.95) | 0.90 (0.87–0.94) |  |
| ≥ 672 cDDDs  (≥96 weeks) | 412 | 50860 | 0.82 (0.75–0.91) | 0.79 (0.71–0.87) |  |
| **All-cause mortality** |  |  |  |  |  |
| Non-users | 49068 | 615204 | 1 [ref] | 1 [ref] |  |
| Overall users | 75330 | 1002956 | 0.93 (0.92–0.95) | 0.93 (0.92–0.94) |  |
| 84-167 cDDDs  (12-24 weeks) | 5265 | 59201 | 1.11 (1.08–1.14) | 1.16 (1.12–1.20) | <.001 |
| 168-335 cDDDs  (24-48 weeks) | 26695 | 319831 | 1.04 (1.02–1.06) | 1.01 (1.00–1.03) |  |
| 336-671 cDDDs  (48-96 weeks) | 39371 | 573064 | 0.85 (0.84–0.86) | 0.86 (0.85–0.87) |  |
| ≥ 672 cDDDs  (≥96 weeks) | 3,999 | 50,860 | 0.98 (0.95–1.01) | 0.90 (0.87–0.93) |  |
| ^a^ Multivariable model was adjusted for age, sex, country of birth, previous clinic visits, previous hospitalization, income levels, education background, Charlson comorbidity index score, family history of CRC, primary ASCVD, regular aspirin use, and the year of index date.  ^b^ *P* for trend was estimated by the log-rank test for trend.  Abbreviations: ASCVD, atherosclerotic cardiovascular disease; cDDD, consecutive defined daily dose; CI, confidence interval; CRC, colorectal cancer; HR, hazard ratio | | | | | |
